# A multicenter prospective observational cohort study to improve diagnostic yield and clinical management of persons with a Bleeding Disorder of Unknown Cause (BDUC-iN study protocol)

**DOI:** 10.1016/j.rpth.2026.106882

**Published:** 2026-07-28

**Authors:** Caroline M.A. Mussert, Amber van Dulmen, Amaury L.L. Monard, Bram Brandt, Floor D.F. Derikx, Nienke Meskes, Tirsa T. van Duijl, Yvonne M.C. Henskens, Maartje van den Biggelaar, Roger E.G. Schutgens, Saskia E.M. Schols, Karin J. Fijnvandraat, Karina Meijer, Paul L. den Exter, Laurens Nieuwenhuizen, Iris van Moort, Marieke J.H.A. Kruip, Ferdows Atiq, Marjon H. Cnossen, Floor C.J.I. Heubel-Moenen

**Affiliations:** 1 Department of Pediatric Hematology and Oncology, Erasmus MC Sophia Children’s Hospital, University Medical Center Rotterdam, Rotterdam, Netherlands; 2 Department of Hematology, Maastricht University Medical Center and Hemophilia Treatment Center Nijmegen-Eindhoven-Maastricht, Maastricht, Netherlands; 3 CARIM—Cardiovascular Research Institute Maastricht, Maastricht University, Maastricht, Netherlands; 4 Department of Molecular Hematology, Sanquin Research, Amsterdam, Netherlands; 5 Central Diagnostic Laboratory, Maastricht University Medical Center, Maastricht, Netherlands; 6 Center for Benign Haematology, Thrombosis and Haemostasis, Van Creveldkliniek, University Medical Center Utrecht, Utrecht, Netherlands; 7 Department Hematology, Radboud University Medical Center, Nijmegen, Netherlands; 8 Hemophilia Treatment Centre Nijmegen-Eindhoven-Maastricht, Nijmegen, Netherlands; 9 Department Pediatric Hematology, Amsterdam UMC Emma Children’s Hospital, University of Amsterdam, Amsterdam, Netherlands; 10 Department of Molecular Cellular Hemostasis, Sanquin Research, Amsterdam, Netherlands; 11 Department Hematology, University Medical Center Groningen, Groningen, Netherlands; 12 Department of Medicine—Thrombosis and Hemostasis, Leiden University Medical Center, Leiden, Netherlands; 13 Department Hematology, Maxima Medical Center, Veldhoven, Netherlands; 14 Department Hematology, Erasmus MC, University Medical Center Rotterdam, Rotterdam, Netherlands

**Keywords:** blood coagulation disorders, hemostasis, prospective studies

## Abstract

**Background:**

In half of individuals referred for evaluation of bleeding symptoms, no hemostatic defect is identified with current standard hemostasis tests. These individuals are classified as having a bleeding disorder of unknown cause (BDUC). The pathophysiological mechanisms underlying the bleeding tendency in BDUC are unknown. The absence of a clear, molecular diagnosis leads to significant challenges for healthcare professionals in terms of diagnostics and clinical management, as well as insecurity in patients and caregivers.

**Objectives:**

The BDUC in the Netherlands (BDUC-iN) study aims to improve diagnostic accuracy and optimize treatment strategies among individuals with BDUC by evaluating healthcare delivery, patient outcomes, health-related quality of life and by identifying underlying pathophysiological mechanisms of the bleeding tendency.

**Methods:**

This is a multicenter prospective observational cohort study aiming to include 500 persons with BDUC. Diagnostic, therapeutic and fundamental research questions are organized in dedicated work packages.

**Conclusions:**

Clinical data is collected longitudinally over a 10-year follow-up period. Quality of life and quality of care are assessed using validated questionnaires. Advanced hemostasis tests and proteogenomics are performed to characterize bleeding phenotypes and potentially identify hemostatic defects.Conclusions: The BDUC-iN study applies a structured approach to address current challenges in BDUC. It aims to improve clinical management and patient outcomes by attaining diagnostic accuracy and developing pathophysiologically-based treatment strategies. In turn, this will support the development of (evidence-based) clinical guidelines and shape future research initiatives in BDUC.

## Essentials

• Bleeding disorder of unknown cause (BDUC) is underrecognized and underresearched.
• The BDUC-iN study addresses challenging diagnostic, therapeutic, and fundamental research questions.
• Gained insights will contribute to improving diagnostic accuracy and clinical management.
• The study offers a collaborative framework and actively welcomes (inter)national partnerships.

## 1 Background and Study Rationale

Despite advances in laboratory diagnostics, current standard hemostasis tests identify a hemostatic defect in only 25% to 50% of individuals referred to a hemostasis specialist for evaluation of bleeding symptoms [1]. Individuals presenting with an increased bleeding tendency, in whom no diagnosis is established with standard laboratory hemostasis tests and other causes are excluded, are classified as having a bleeding disorder of unknown cause (BDUC) [[1], [2], [3]]. The observed bleeding symptoms are overall similar to that of individuals with a mild to moderate bleeding disorder, for example, von Willebrand disease or a platelet function disorder, with bleeding symptoms predominantly mucocutaneous, such as hematomas, epistaxis, and menorrhagia, as well as excessive bleeding after medical and dental procedures and childbirth [[2], [3], [4], [5], [6], [7]].

Although the International Society on Thrombosis and Haemostasis (ISTH) Scientific and Standardization Committee (SSC) has published recommendations for BDUC [8], no standardized guidelines exist for diagnosis and clinical management, mainly due to clinical heterogeneity and limited knowledge regarding the diverse pathophysiology of the bleeding tendency. The ISTH SSC has proposed a minimal panel of standard hemostasis tests, with additional tests depending on local preferences and test availability [8]. This, of course, leads to a persisting large variability in current clinical practice [9]. Besides standard hemostasis tests, new and more advanced hemostasis laboratory tests, such as thrombin generation, viscoelastic testing, and genetic testing, have been performed in research settings to investigate BDUC-underlying pathophysiological mechanisms [[10], [11], [12], [13], [14], [15], [16], [17]]. Nevertheless, their added value remains unclear due to limited availability, inconsistent results, and low diagnostic yield, as well as variation in methodology complicating comparison of results. Emerging approaches such as proteomics [18], flow-based hemostatic assays [12], and tests assessing the fibrinolytic system [19,20], potentially offer discriminatory value. As BDUC likely is a heterogeneous condition with multiple underlying biological mechanisms, no single laboratory assay is expected to explain the bleeding phenotype in all patients. Therefore, the systematic investigation of complementary domains of hemostasis, including coagulation, fibrinolysis, platelet function, endothelial function, and molecular determinants of hemostasis, with use of advanced hemostasis tests, is essential.

The absence of a clear diagnosis poses significant challenges when managing patients with BDUC clinically during hemostatic challenges, for example, invasive procedures, and childbirth. Overall persons with BDUC are managed according to a general treatment plan, often consisting of tranexamic acid and/or desmopressin [4,21,22]. However, minor and major bleeding still occurs despite preventive measures, suggesting that treatment is not always adequate [4,7,22]. Moreover, nonhematological factors may also contribute to bleeding symptoms. In the absence of pathophysiologically based treatment strategies, undertreatment and overtreatment is common, such as unnecessary use of blood products or hemostatic agents, leading to excessive bleeding risks, complications, and/ or costs [4,21]. Therefore, development of more targeted and effective treatment strategies is urgently needed.

From a patient’s perspective, major but also nuisance bleeding significantly impacts social functioning, school, academic and occupational performance, impairing health-related quality of life (HRQoL), especially in women with heavy menstrual bleeding (HMB) [23,24]. While research on HRQoL in BDUC is limited, 1 study reported reduced physical and mental HRQoL in individuals with BDUC [24]. Furthermore, a BDUC diagnosis raises uncertainties regarding bleeding risk, clinical management around child delivery, and genetic counseling [21]. Although a positive family history may be present, genetic background of bleeding in affected families is often unexplored, complicating risk assessment in families. Evaluating patient experiences regarding BDUC diagnosis and current care delivery is essential to identify unmet needs and guide improvements in the clinical management and support of persons with BDUC.

To address these many unmet needs in BDUC, the BDUC in the Netherlands (BDUC-iN) working group was established, in close collaboration with the SYMPHONY consortium [25]. In the BDUC-iN working group, all treatment centers for rare bleeding disorders in the Netherlands and Sanquin Research are represented. In preparation for the national BDUC-iN study, the working group published an illustrated review outlining previous research and current knowledge gaps, highlighting directions with outstanding research questions in BDUC [26]. Moreover, a survey was published investigating current practices regarding BDUC in the Netherlands [9]. In addition, retrospective data from 3 existing national BDUC cohorts were merged to explore clinical and laboratory characteristics in a larger patient population. Together, this provided the rationale for the prospective BDUC-iN study, aiming to address ongoing diagnostic, therapeutic, and fundamental research questions concerning the pathophysiological mechanisms underlying the bleeding tendency observed in persons with BDUC.

### 1.1 Study objectives

The aim of the BDUC-iN study is to identify and evaluate health care delivery, patient outcomes, and HRQoL in persons with BDUC (objective A). In addition, we aim to investigate the pathophysiological mechanisms underlying bleeding tendency and to identify subgroups in BDUC based on clinical and laboratory parameters (objective B). Lastly, the study aims to improve the diagnostic yield and optimize treatment strategies for persons with BDUC (objective C).

## 2 Patients and Methods

### 2.1 Study design and work packages

**Figure 1:**
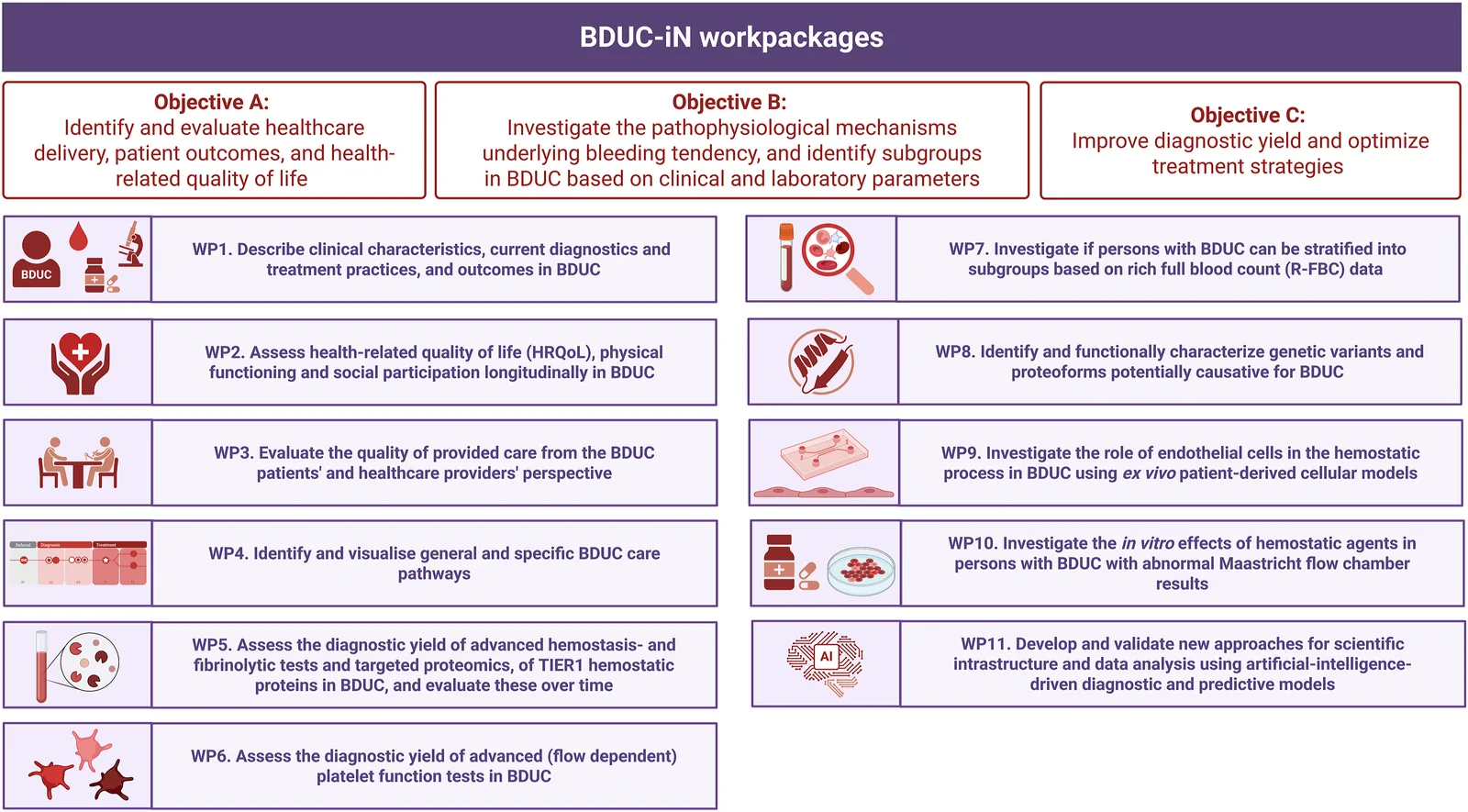
Overview of BDUC-iN study workpackages (WPs). BDUC, bleeding disorder of unknown cause; BDUC-iN, BDUC in the Netherlands.

The BDUC-iN study is designed as a multicenter, prospective observational cohort study. The study will be conducted across all treatment centers for rare bleeding disorders in the Netherlands in collaboration with Sanquin Research (HemoTwin consortium), with the Maastricht University Medical Center serving as the coordinating site. The BDUC-iN study will be performed in close collaboration with the Dutch Society for Hemophilia Treaters (NVHB), Dutch Hemophilia Nursing Society (NVHV), Netherlands Society of Hemophilia Patients (NVHP), and the SYMPHONY consortium [25]. The BDUC-iN study will therefore bring together many different disciplines including health care professionals (HCPs); fundamental scientists in molecular and cellular biology; multiomics scientists (genomics and proteomics); data analysts; biomedical statisticians; health economists; HRQoL experts; and patients and their representatives. To achieve the study objectives, research questions are organized in dedicated workpackages (WPs) that are outlined further and summarized in Figure 1.

#### 2.1.1 Workpackage 1

The objective is to describe clinical characteristics, current diagnostics and treatment practices, and outcomes in BDUC. In WP1, we will collect clinical data on diagnosis, treatment, and clinical follow-up longitudinally over a 10-year period from electronic patient records, patient interviews, and laboratory assessments. The following end points will be evaluated: (1) (general) patient characteristics; (2) prevalence and incidence of bleeding; (3) incidence of hypermobility; (4) time to diagnosis, reasons for referral, and performed diagnostic tests; (5) treatment plans and outcomes in cases of bleeding, medical interventions, and childbirth; and (6) longitudinal course of bleeding phenotype and treatment response. The natural history of the bleeding symptoms over time and treatment outcomes will be analyzed to identify longitudinal trends, treatments effects, and patterns in disease progression or stabilization.

#### 2.1.2 Workpackage 2

The objective is to assess HRQoL, physical functioning, and social participation longitudinally in BDUC patients. In WP2, we will collect data on HRQoL, physical functioning, and social participation using validated questionnaires. Particular attention will be given to the prevalence and impact of HMB on HRQoL in women and girls. Questionnaires (see also Section 3.3) will be completed by participants at study inclusion and at 5-year and 10-year follow-up intervals. This will facilitate the identification of trends over time, as well as the assessment of the physical and emotional impact of clinical events (eg, new bleeding episodes, surgery). Based on (preliminary) results, participants may be asked to complete additional (optional) questionnaires.

#### 2.1.3 Workpackage 3

The objective is to evaluate the quality of provided care from the BDUC patients’ and health care providers’ perspective. In WP3, we will perform semistructured interviews and focus groups in a subgroup of patients and HCPs to gain in-depth insights into the experiences and needs of individuals and their HCPs regarding the current care provision. Discussed topics will include experiences and needs regarding the diagnostic trajectory and its impact, provided information, communication with HCPs, accessibility and continuity of care, and provided treatment. A comprehensive thematic content analysis including several phases of coding will identify areas for improvement and enable the provision of more patient-centered care.

#### 2.1.4 Workpackage 4

The objective is to identify and visualize general and specific BDUC care pathways. Available clinical guidelines and documentation and interactive cocreation sessions with HCPs will be used to identify current care processes. The care pathway will then be visualized using the Metro Mapping methodology [27]. Mapping the BDUC care pathway facilitates standardization of care, highlights existing gaps or redundancies, and identifies opportunities for health care optimization. Longitudinal data collected in WP1, WP2, and WP3 will be integrated into the constructed pathways to identify data-driven areas of improvement, indicating where clinical decision making is variable, care is suboptimal, or additional guidance is needed.

#### 2.1.5 Workpackage 5

The objective is to assess the diagnostic yield of advanced hemostasis and fibrinolytic tests and targeted proteomics of TIER1 hemostatic proteins in BDUC and evaluate these over time. In WP5, we will perform advanced hemostasis and fibrinolytic tests eg, assessment of rare factors deficiencies, modified thrombin generation assays and tissue plasminogen–activated rotational thromboelastometry, to assess their diagnostic yield in BDUC. Previous studies demonstrated hyperfibrinolytic profiles in a subset of persons with BDUC, supporting further evaluation of fibrinolytic pathways using tissue plasminogen–activated rotational thromboelastometry [20]. Moreover, targeted proteomics will be performed according to the ISTH-Tier1 gene list [28,29]. These assays aim to elucidate abnormalities in coagulation and fibrinolytic pathways. To assess intraindividual changes over time, blood samples will be collected at 5 and 10 years after study inclusion for longitudinal evaluation of hemostatic parameters. Particular attention will be given to the well-known age-related increase in von Willebrand factor (VWF) levels [30], and its impact on diagnostic reclassification and bleeding phenotype.

#### 2.1.6 Workpackage 6

The objective is to assess the diagnostic yield of advanced (flow dependent) platelet function tests in BDUC. In WP6, we will assess the diagnostic yield of advanced (flow-dependent) platelet function tests (eg, the Maastricht flow chamber [12], β-thromboglobulin, and platelet serotonin content). A subset of participants with a possible platelet function disorder will be reinvited for an additional blood withdrawal to perform platelet proteomics at Sanquin Research to investigate for qualitative and/or quantitative defects in the platelet proteome [31,32].

#### 2.1.7 Workpackage 7

The objective is to investigate whether persons with BDUC can be stratified into subgroups based on rich full blood count (R-FBC) data. When complete blood counts are measured in fluorescence platelet count modus, the Sysmex hematology analyzer produces an extended dataset, including, for example, which is referred to as R-FBC. In WP7, we will measure complete blood counts in fluorescence platelet count modus, including, for example, the immature platelet fraction, immature reticulocyte fraction and platelet large cell ratio, also referred to as R-FBC. In WP7, using R-FBC data, persons with BDUC will be stratified into subgroups based on platelet parameters, hemoglobin parameters, and leucocyte parameters.

#### 2.1.8 Workpackage 8

The objective is to identify and functionally characterize genetic variants and proteoforms potentially causative for BDUC. In WP8, we will investigate whether multiomics analysis can unravel the biological mechanisms underlying the bleeding phenotype in BDUC. A whole exome sequencing hemostasis and thrombosis gene panel test [33] will be performed in participants who consented. Adult participants with a severe bleeding phenotype (ISTH–bleeding assessment tool [BAT] > 8) and preferably a positive family history for bleeding, will be invited for genetic counseling by a clinical geneticist and asked additional informed consent to perform open exome analysis and whole genome sequencing (WGS). In addition, unbiased proteomics by quantitative liquid chromatography-tandem mass spectrometry/mass spectrometry will be performed, to explore the potential involvement of alternative proteins in hemostatic disbalance, such as the complement system or endothelial cell markers. Within the HemoTwin consortium, genomic data will be integrated with proteomic data, to compare findings from patients with a known genetic bleeding disorder enrolled in previous Dutch national cohort studies, such as the Hemophilia in the Netherlands (HiN) study [34], Willebrand in the Netherlands (WiN) study [35], Thrombocytopathy in the Netherlands (TiN) study [36], and Rare Bleeding Disorders in the Netherlands (RBiN) study [37].

#### 2.1.9 Workpackage 9

The objective is to investigate the role of endothelial cells in the hemostatic process in BDUC using *ex vivo* patient-derived cellular models. Dysfunction of endothelial cells or their interaction with blood cells, such as platelets and hemostatic proteins, may contribute to the observed bleeding phenotype. In WP9, we will therefore study secretory mechanisms of endothelial cells and platelets to identify new determinants of hemostasis and vascular health. This exploratory model is intended for mechanistic hypothesis generation and not for diagnostic classification or clinical decision making. *Ex vivo* (patient-derived) cellular model systems for endothelial and platelet secretion will be developed and integrated into an innovative microfluidic vessel-on-a-chip [38]. This will enable real-time imaging of blood flow and visualization of the endothelium, platelets, and specific plasma proteins. Furthermore, a controlled vascular injury can be introduced via a microengineered pneumatic valve, allowing for real-time observation and analysis of the hemostatic response [39].

#### 2.1.10 Workpackage 10

The objective is to investigate the *in vitro* effects of hemostatic agents in persons with BDUC with abnormal Maastricht flow chamber results. In WP10, we aim to investigate whether the addition of hemostatic agents that support hemostasis (tranexamic acid, VWF/factor [F]VIII concentrates, desmopressin, and platelets) is able to normalize hemostasis *in vitro* in persons with BDUC and abnormal Maastricht flow chamber results. A subgroup of participants presenting with a severe bleeding phenotype (defined as an ISTH-BAT score > 8) and abnormalities in the Maastricht flow chamber will be selected for an additional blood withdrawal. The Maastricht flow chamber will be performed on these samples before and after addition of various hemostatic agents. Key parameters that will be evaluated include platelet adhesion, activation, and aggregation; collagen/tissue factor–mediated thrombus formation, and fibrin formation under venous and arterial shear rate over time.

#### 2.1.11 Workpackage 11

The objective is to develop and validate new approaches for scientific infrastructure and data analysis using artificial intelligence–driven diagnostic and predictive models. In WP11, clinical, laboratory, and genetic data will be integrated into a mixed-variable dataset and analyzed collectively. Unsupervised clustering techniques and machine learning techniques will be applied to identify biologically and clinically relevant patient subgroups. Identified clusters will subsequently be characterized based on bleeding phenotype, laboratory abnormalities, and treatment outcomes. Moreover, this stratification will enable comparative analysis of clinical outcomes and treatment responses across identified subgroups. Artificial intelligence–driven algorithms and bleeding prediction models will be developed and validated using machine learning techniques, clinical patient profiles, and biomarker data. In parallel, we will explore the feasibility of establishing a national bioresource facility in the Netherlands. This will involve evaluating existing international models, such as those in the United Kingdom [40], and explore how such frameworks can be adapted and implemented.

### 2.2 Inclusion and exclusion criteria

Inclusion and exclusion criteria are presented in Table 1 [41]. Inclusion criteria are aged ≥ 12 years; referred to a (pediatric) hemostasis specialist for evaluation of bleeding tendency; increased bleeding tendency based on an abnormal ISTH-BAT score or based on clinical gestalt according to the investigating physician; and an absence of diagnostic test results for a bleeding disorder in standard laboratory hemostasis tests (Table 1) [8].

**Table 1:** Inclusion and exclusion criteria.

**Inclusion criteria**	
Age ≥12 y	
Referred to a (pediatric) hemostasis specialist for evaluation of bleeding tendency	
Increased bleeding tendency based on: - Abnormal ISTH-BAT score (≥5 in women aged 18-30 y, ≥6 in women aged 31-51 y, and ≥7 in women age 52 y or older; ≥4 in men; and ≥ 3 in children) [41] OR - Clinical gestalt according to the investigating physician	
Absence of diagnostic test results for a bleeding disorder in standard laboratory hemostasis tests:	
Laboratory test	For inclusion should be
Complete blood count	Hemoglobin > 6.0 mmol/L; thrombocyte count > 100 × 10^9^/L
PT, aPTT	Within local reference range, or prolonged without explanatory factor deficiency
Fibrinogen activity; VWF antigen and activity; and FVIII, FIX, FXI, and FXIII	Within local reference range or abnormal but not explaining bleeding phenotype
Light transmission aggregometry	Not diagnostic for a platelet function disorder or not explaining bleeding phenotype
Kidney function: EGFR	> 45 mL/min
Liver function: ALAT, bilirubin	< 3× ULN
**Exclusion criteria**	
Use of medication interfering with laboratory hemostasis tests, which cannot be stopped before blood withdrawal (eg, anticoagulant or antiplatelet therapy and NSAIDs)	
Pregnancy or lactation at moment of inclusion	
Presence of a known bleeding disorder	
Presence of an acquired cause or another explanation for the increased bleeding tendency	
Inability to provide informed consent	

ALAT, alanine aminotransferase; aPTT, activated partial thromboplastin time; EGFR, estimated glomerular filtration rate; F, factor; ISTH-BAT, International Society on Thrombosis and Haemostasis—Bleeding Assessment Tool; NSAID, nonsteroidal anti-inflammatory drug; PT, prothrombin time; VWF, von Willebrand factor.

A potential participant will be excluded in case of use of medication interfering with laboratory hemostasis tests, which cannot be stopped before blood withdrawal; pregnancy or lactation at moment of inclusion; presence of an established bleeding disorder [[42], [43], [44]]; presence of an acquired cause or another explanation for the increased bleeding tendency; or inability to provide informed consent.

## 3 Study Procedures

**Figure 2:**
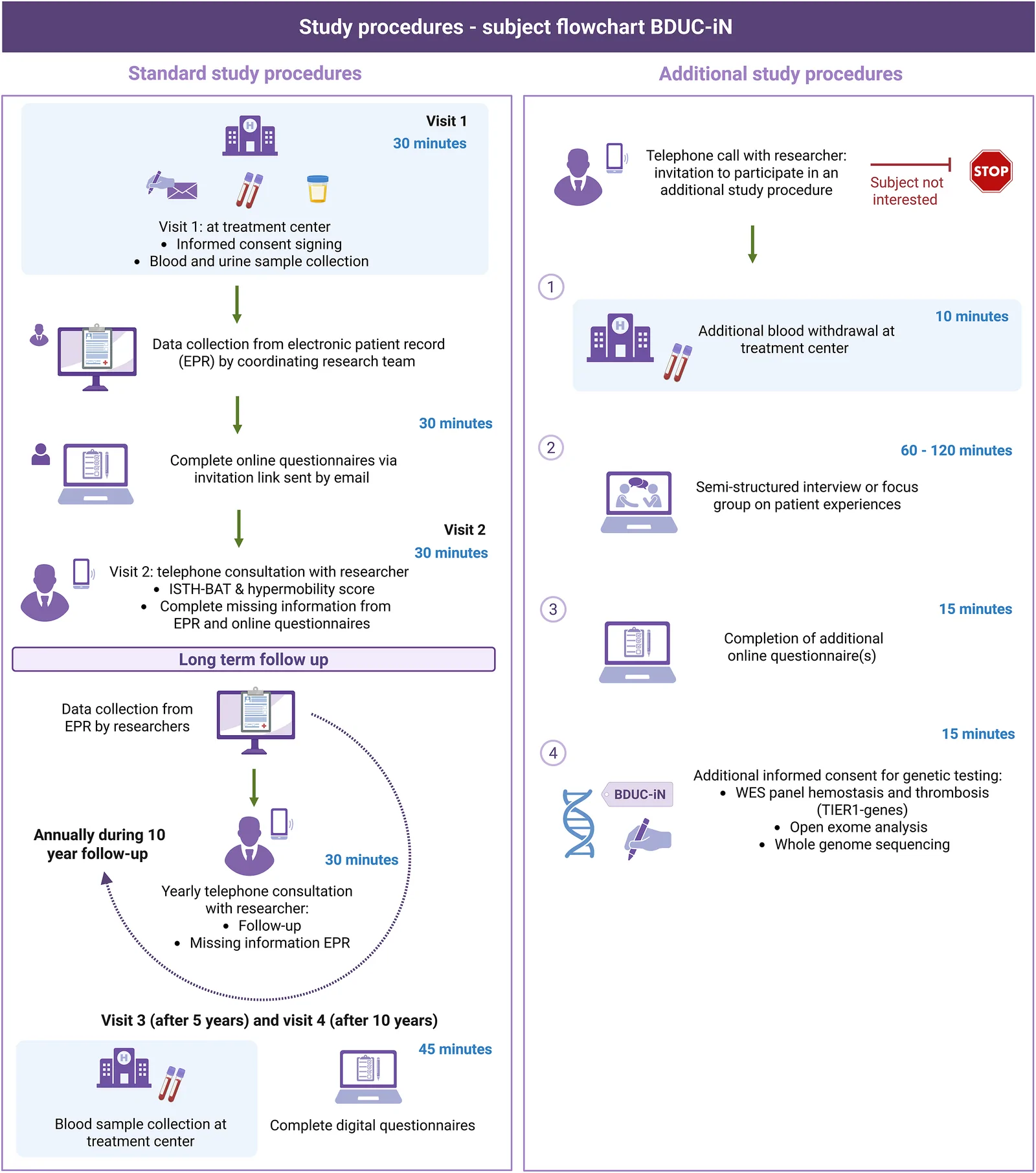
Overview of study procedures. BDUC, bleeding disorder of unknown cause; EPR, electronic patient record; ISTH-BAT, International Society on Thrombosis and Haemostasis—Bleeding Assessment Tool; WES, whole exome sequencing.

A mixed-methods approach is used for data collection and analysis. An overview of the study procedures is shown in Figure 2. If inclusion and exclusion criteria are met and the person agrees to participate in the study, informed consent is signed at the start of visit 1. Consent is asked for study inclusion and for reinvitation for additional study procedures. Informed consent for DNA analysis (whole exome sequencing hemostasis and thrombosis gene panel) is asked from all participants in a separate appendix of the participant information form.

During visit 1, blood samples and a urine sample are collected. After this visit, information is obtained from the electronic patient record (EPR) by the research team, and questionnaires are sent digitally or on paper to participants to be completed at home. After ∼2 months, a telephone consultation is scheduled (visit 2). During this visit, the ISTH-BAT and hypermobility are scored by a trained and experienced researcher. Moreover, missing information from the EPR and online questionnaires are completed. Follow-up consists of an annual telephone consultation and check of the EPR by the study team to collect data on bleeding symptoms, ISTH-BAT score, hemostatic challenges, treatment, and outcomes. At 5 and 10 years after inclusion, another visit to the treatment center is scheduled (visit 3 and visit 4, respectively) to collect an additional blood sample, and digital questionnaires are repeated. Treatment center visits and blood collection are coordinated with routine clinical visits or scheduled venipunctures if possible to minimize participant burden.

### 3.1 Blood and urine collection

Visit 1 is scheduled in the morning and participants are instructed to eat only a light, nonfat containing breakfast. Medication known to interfere with laboratory tests is discontinued for at least 10 days prior to testing. During visit 1 a maximum of 100 mL of blood and 10 mL of urine is collected from each participant. These samples are either stored or immediately processed for advanced hemostasis testing, platelet function tests, targeted proteomics, assessment of vascular markers, and, if consent has been provided, DNA gene panel testing for undiagnosed bleeding and platelet disorders. Not all investigations are performed in all participants because some tests require fresh whole blood and can only be performed in centers where this test is available (eg, ATP release and Maastricht flow chamber). If a test has already been performed in the participating center before BDUC diagnosis, the test is not repeated. Performed tests are shown in Table 2. After 5 and 10 years of follow-up, an additional blood sample (30 mL) is collected for VWF activity and antigen, FVIII, thrombin generation, and plasma proteomics among others.

**Table 2:** Performed blood tests at baseline and follow-up visits^a^.

**(Advanced) hemostasis tests at baseline**	
Rare coagulation factor deficiency	FII, FV, FVII, and FX
Hemophilia/VWD	VWF activity and antigen, VWF collagen binding, 1-stage and chromogenic FVIII, and ABO blood group
Fibrinolysis disorders	Fibrinogen, PAI-1 activity and antigen, tPA, plasminogen, TAFI, α2-AP, lysis timer test, ECLT, plasmin generation test, and tPA-ROTEM
Anticoagulant abundance	D-dimer, antitrombin, protein C, protein S, and TFPI
Global hemostasis (dys)function	Thrombin generation^b^ and NATEM-ROTEM
Other causes for bleeding	TSH, ALAT, creatinine, EGFR, and vitamin C
Proteomics and genetic testing (if consent is present)	Targeted and unbiased proteomics^b^ and WES panel hemostasis and thrombosis (TIER-1 genes)
**(Advanced) tests for platelet function disorders at baseline**	
Global platelet function tests	Complete blood count in PLT-F modus with immature platelet fraction
Platelet nucleotide assessment	ATP-release, ATP/ADP, platelet serotonin content, and β-TG
Flow-based platelet function tests	Maastricht flow chamber^b^, T-TAS^b^, Anysis^b^, and PFA-200
Flowcytometry	VWF, GP1b, CD49b, CD41a, and GPVI
Platelet activation test (PACT)	PAR1, PAR4, ADP, CRP, and U46619 for fibrinogen/p-selectin expression
**Longitudinalfollow-upat 5 and 10 y**^c^	
Hemophilia/VWD	FVIII and VWF antigen and activity
Global hemostasis (dys)function	Thrombin generation^b^
Proteomics	Plasma proteomics^b^

ALAT, alanine aminotransferase; AP, antiplasmin test; ECLT, euglobulin clot lysis time; EGFR, estimated glomerular filtration rate; NATEM, nonactivated rotational thromboelastometry; PAI, plasminogen activator inhibitor; PFA, platelet function analyzer; PLT-F, fluorescence platelet count; ROTEM, rotation thromboelastometry; tPA, tissue-type plasminogen activator; TAFI, thrombin-activatable fibrinolysis inhibitor; TFPI, tissue factor pathway inhibitor; TG, thromboglobulin; TSH, thyroid-stimulating hormone; T-TAS, total thrombus formation analysis system; VWD, von Willebrand disease; VWF, von Willebrand factor.

a If a test has already been performed in the participating center, the test is not repeated.

b Research-based tests.

c Based on preliminary results and new insights, additional assessments may be added to follow-up evaluations.

### 3.2 Data collection from EPRs

EPRs are reviewed to collect data on demographic and socioeconomic characteristics, as well as clinical data such as reasons for initial referral, bleeding symptoms, a medical history, a family history, medication use, contraceptive use, and the diagnostic pathway leading to BDUC diagnosis, including BAT score and performed laboratory tests at time of BDUC diagnosis. Additionally, details regarding treatment plans and outcomes related to invasive procedures, bleeding episodes, and childbirth within the past 5 years are collected. This includes type of intervention and associated outcomes (eg, bleeding, re-surgery, prolonged hospital stays, and use of hemostatic agents and blood products). These data are also documented throughout the follow-up period by annual review of the EPR.

### 3.3 Online questionnaires

Standardized questionnaires are administered to collect data on sociodemographic details, physical activity, mental health, social participation, and, in women and girls, HMB-specific QoL. Adult participants will complete the short form (SF)/RAND-36 questionnaire, the generic patient-reported outcome measure (PROM) questionnaire, and the short questionnaire to assess health-enhancing physical activity (SQUASH) and, for women, additionally, the menorrhagia multiattribute scale (MMAS). Children will complete the pediatric quality of life inventory (PEDsQL), PROM pediatric measures, and SQUASH, and, for girls, additionally, the MMAS.

### 3.4 Additional study procedures

A selection of study participants, if consent to be reinvited is present, will be invited for additional study procedures (Figure 2), based on specific clinical or laboratory characteristics and results from WPs. These additional procedures include an additional blood withdrawal to perform advanced hemostasis tests (WP5 and WP6), flow chamber experiments (WP10), multiomics analysis (WP8), and endothelial cell models (WP9); participation in semistructured interviews or focus groups to explore patient experiences and needs regarding BDUC (WP3); completion of additional online questionnaires (WP2); and for adults (age ≥ 18, including those who reach adulthood during the course of the study), genetic counseling and additional informed consent for open exome analysis and WGS (WP8).

### 3.5 Sample size

The BDUC-iN study consists of multiple workpackages with distinct objectives and end points. Therefore, no single conventional power calculation based on 1 primary outcome was considered appropriate. The planned sample size was primarily based on feasibility and the expected precision of estimating the diagnostic yield of advanced hemostatic testing in BDUC. Previous studies and preliminary data suggest that extended laboratory investigations may identify clinically relevant abnormalities in ∼20% of individuals currently classified as having BDUC [20,45]. Assuming a diagnostic yield of 20%, inclusion of 500 participants allows estimation of this proportion with a 95% confidence interval of approximately ±3.5%. Furthermore, this sample size is expected to yield ∼100 participants with an abnormal laboratory phenotype, facilitating exploratory subgroup analyses across the different workpackages. Given the rarity and heterogeneity of BDUC, inclusion of 500 participants is considered both feasible and scientifically meaningful. Within 3 existing Dutch cohort studies, ∼300 individuals with BDUC are currently registered, who will be invited for inclusion in the BDUC-iN study. Willingness to participate was high in prior studies. As the incidence of BDUC in the Netherlands is estimated at ∼50 to 100 new cases per year, a total enrolment of ∼500 participants over the entire study period is considered realistic.

### 3.6 Ethics and dissemination

The BDUC-iN study protocol was approved by the Medical Ethical Research Committee of the Maastricht University Medical Center, Maastricht, Netherlands (NL87370.068.24), and registered at ClinicalTrials.gov (NCT07454161) and the Overview of Medical Research in the Netherlands (NL-OMON57561). Approval by the local Medical Ethical Research Committees of participating treatment centers is currently under review. All amendments to the protocol will be submitted to the Medical Ethical Research Committee and to the competent authority.

Study procedures are conducted in accordance with the principles of the Declaration of Helsinki and the Medical Research Involving Human Subjects Act (WMO). Informed consent is obtained from all participants for study participation with use of data for research purposes. In case of open and WGS, there is a risk of incidental findings. Participants and their treating physicians are informed about unsolicited findings that require follow-up care or in case of relevance for their offspring.

Participant enrollment will start on March 1, 2026, with an inclusion period of up to 5 years. All study participants will subsequently be followed up for a 10-year period. Results from the different participating treatment centers will be analyzed in close collaboration with these centers and submitted for publication in international peer-reviewed journals as well as presented at scientific conferences. Depending on the research question, 1 or more participating centers will take the lead in the analysis of results and preparation of the manuscripts for publication, after approval by the principal investigator and the BDUC-iN steering committee.

## 4 Conclusion

Despite the considerable clinical burden, BDUC remains poorly investigated and underrecognized in clinical practice. The lack of identifiable underlying hemostatic defects complicates diagnostic evaluation and impedes the development of evidence-based, personalized treatment plans. The BDUC-iN study aims to create a comprehensive and structured approach to address unmet needs with an interdisciplinary research proposal. The pathophysiological mechanisms underlying the clinical bleeding phenotype in BDUC will be systematically analyzed, while simultaneously evaluating health care delivery, patient outcomes, and HRQoL. By integrating diagnostic, therapeutic, and fundamental objectives, the BDUC-iN study aims to improve diagnostic accuracy and support the development of pathophysiologically based treatment strategies. In turn, this will enhance patient outcomes and quality of care for individuals with BDUC, improving patient-centered care. Furthermore, insights from this study will contribute to increased awareness of BDUC among health care providers and direct future guidelines and research. The BDUC-iN study offers a collaborative framework and actively welcomes national and international partnerships aimed at advancing mechanistic insights, diagnostic innovation and evidence-based management of BDUC. Publication of this protocol aims to enhance transparency, facilitate international collaboration, and harmonize future BDUC research efforts.
